# Orexinergic neurotransmission in temperature responses to amphetamines

**DOI:** 10.1186/1471-2202-14-S1-P430

**Published:** 2013-07-08

**Authors:** Yaroslav I Molkov, Daniel Fu, Patrick Tan, Maria V Zaretskaia, Dmitry V Zaretsky

**Affiliations:** 1 Department of Mathematical Sciences, Indiana University - Purdue University Indianapolis, IN 46202, USA; 2 Park Tudor School, Indianapolis, IN 46240, USA; 3 Carmel High School, Carmel, IN 46032, USA; 4 Department of Emergency Medicine, Indiana University School of Medicine, Indianapolis, IN 46202, USA

## 

Derivatives of amphetamines are widely abused all over the world. After long-term use cognitive, neurophysiological, and neuroanatomical deficits have been reported. Neurophysiological deficits are enhanced by hyperthermia, which itself is major mortality factor in drug abusers. Temperature responses to injections of methamphetamine are multiphasic and include both hypothermic and hyperthermic phases, which are highly dependent on ambient temperature and previous exposure to the drug. Also, amphetamine derivatives differentially affect various neuromediator systems, such as dopaminergic, noradrenergic, serotonergic. Finally, body temperature is dependent on multiple thermoregulatory mechanisms and complex neuronal circuitry.

Temperature responses to methamphetamine (Meth) at room temperature have non-trivial dose-dependence, which is far from being understood. Intermediate doses of Meth cause less hyperthermia than both low and high doses of the drug. Also, maxima of all responses have different latency: responses to low and high doses are virtually immediate, while a response to an intermediate dose appears to be delayed. In our previous modeling study we demonstrated that such dose-dependence can be explained by interaction of inhibitory and excitatory drives induced by Meth [1]. Recently, we have published data on the involvement of orexinergic neurotransmission in Meth-induced temperature responses [2] where the low dose (10 mg/kg, i.p.) of SB-334867, an antagonist of the first type of orexin receptors (ORX1), was injected 30 min prior to various doses of Meth. While this dose of antagonist clearly suppressed the response to low (1 mg/kg) and intermediate (5 mg/kg) doses of Meth, the effect was statistically significant only at the late phase (t>60 min) of the response to intermediate dose. At the early phase (t<60 min) any drug-related changes were marred by stress-induced temperature fluctuations resulting from two intraperitoneal injections.

In a separate set of experiments, which is reported here for the first time, a high dose of the same antagonist (30 mg/kg, i.p.) suppressed the effect of low doses of Meth even more, but in contrast, it significantly amplified the responses to the higher doses (5 and 10 mg/kg) of Meth. Since resolving this seeming controversy can have profound importance by explaining cases of malignant hyperthermia after "physiological" doses of Meth, we performed a mathematical modeling study to provide its mechanistic interpretation. We hypothesize that a specific distribution of orexin receptors over the structures involved in the neural control of temperature is responsible for this complex dependence of the Meth-induced responses on the dose of orexin antagonist. To uncover this distribution we extend our previous model [1] that allowed us to reconstruct the activation curves of the Meth-sensitive compartments and dose dependence of the dynamics of the neural populations involved and to reproduce body temperature responses for various doses of Meth. We use the extended model to elucidate the role of orexinergic neurotransmission in dose-dependent temperature responses to Meth.
